# Climate change: Does international research fulfill global demands and necessities?

**DOI:** 10.1186/s12302-020-00419-1

**Published:** 2020-10-15

**Authors:** Doris Klingelhöfer, Ruth Müller, Markus Braun, Dörthe Brüggmann, David A. Groneberg

**Affiliations:** 1 Institute of Occupational, Social and Environmental Medicine, Goethe University, Theodor-Stern-Kai 7, 60590 Frankfurt, Germany; 2 Unit Entomology, Institute of Tropical Medicine, Nationalestraat 155, 2000 Antwerp, Belgium

**Keywords:** Global warming, Greenhouse effect, Bibliometrics, Socioeconomic indices, Climate inequity, Research investment

## Abstract

**Background:**

Climate change is safe to be one of the biggest challenges of mankind. Human activities, especially the combustion of fossil fuels, contribute to the increase of greenhouse gases in the atmosphere and thus to the pace of climate change. The effects of climate change are already being felt, and the resulting damage will most likely be enormous worldwide. Because global impacts vary widely and will lead to very different national vulnerability to climate impacts, each country, depending on its economic background, has different options to ward off negative impacts. Decisions have to be made to mitigate climate consequences according to the preparedness and the vulnerability of countries against the presumed impacts. This requires a profound scientific basis. To provide sound background information, a bibliometric study was conducted to present global research on climate change using established and specific parameters. Bibliometric standard parameters, established socioeconomic values, and climate change specific indices were used for the analyses. This allowed us to provide an overall picture of the global research pattern not only in terms of general aspects, but also in terms of climate change impacts, its effects and regional differences. For this purpose, we choose representative indices, such as the CO_2_ emissions for the responsibility of countries, the global climate risk index as a combination value for the different types of damage that countries can expect, the increase in sea level as a specific parameter as a measure of the huge global environmental impacts, and the readiness and vulnerability index for the different circumstances of individual countries under which climate change will take place. We hope to have thus made a comprehensive and representative selection of specific parameters that is sufficient to map the global research landscape. We have supplemented the methodology accordingly.

**Results:**

In terms of absolute publication numbers, the USA was the leading country, followed by the UK, and China in 3rd place. The steep rise in Chinese publication numbers over time came into view, while their citation numbers are relatively low. Scandinavian countries were leading regarding their publication numbers related to CO_2_ emission and socioeconomic indices. Only three developing countries stand out in all analyses: Costa Rica, the Fiji Atoll, and Zimbabwe, although it is here that the climate impact will be greatest. A positive correlation between countries’ preparedness for the impacts of climate change and their publication numbers could be shown, while the correlation between countries’ vulnerability and their publication numbers was negative.

**Conclusions:**

We could show that there exists an inequity between national research efforts according to the publication output and the demands and necessities of countries related to their socioeconomic status. This inequity calls for a rethink, a different approach, and a different policy to improve countries' preparedness and mitigation capacity, which requires the inclusion of the most affected regions of the world in a strengthened international cooperation network.

## Background

Particularly in the western world, public awareness of the consequences of climate change has reached a high level. Before the appearance of the coronavirus pandemics (SARS-CoV 2), hardly any news broadcast in the western world could do without commentary on climate change. Every week millions of pupils and students around the world demonstrated all for a strict ecological regimen of all governments to ensure the 2 °C target of the Paris Agreement [42]. In “Corona times” the effects of climate change seem almost forgotten by the public, although many scientists have already explained the connection between climate change and the increase in zoonoses [24, 36]. Besides, the negative effects of climate change will certainly be more permanent and severe than the temporary damage of a pandemic; however, severe it may be.

Climate change will undoubtedly affect the entire planet and calls for international collective action. Shifts in wind patterns, the average temperature, or the amount of precipitation and frequency of extreme weather events will endanger the health, the food, and the water supply for humans. Those risks are directly linked to the reduction in biological diversity and the extinction of species that challenge most parts of the world. The impacts of climate change will lead to socioeconomic and political instability, which will change the living conditions of many communities.

The global climate has always been changing. However, the enormous problems are caused by the speed with which changes due to human intervention are progressing, and greenhouse gas concentrations have reached levels never before experienced by mankind. Although climate change has officially been considered the most hazardous global risk so far, the recent Conference of Parties (COP) in Madrid failed to achieve binding measures for nations.

But time is running. Solutions must be found to mitigate the consequences of climate change. Governments must react and be prepared for the worst future scenarios that require strategies without national borders. Climate change affects every country in different ways, and the ways in which countries can prepare for it or mitigate its impacts vary widely.

But what has actually happened so far? Anthropogenic activities, in particular the combustion of fossil fuels, have accelerated the rise in carbon dioxide emissions and thus the increase in global warming, with tangible impacts on humans, animals, and the ecological balance around the world [45]. The immediate environmental consequence of global warming is the increase in natural disasters, e.g., melting glaciers, more extreme and more frequent floods, wildfires, storms, and droughts or heatwaves. The indirect consequences include threats to human health, and the reduction of biodiversity and habitable areas, leading to migration and deterioration of community, public health, and socioeconomic conditions in most countries of the world [45].

Reliable estimation of the extent of these impacts is at the heart of research and forms the basis for all mitigation strategies at governmental, economic, scientific, or personal levels.

A sound research database is necessary for sustainable approaches for assessing and mitigating climate change impacts. The research on the climate change focuses on a wide range of areas and modeling approaches to consider different future carbon dioxide (CO_2_) emission scenarios to assess local and regional global warming. CO_2_ is a major component of the global carbon cycle and both a natural part of the atmosphere and an essential greenhouse gas. It is mainly through the combustion of fossil fuel that humans influence the amount of CO_2_ emission and thus contributes to global warming.

For this, experts who cover all areas of climate change are in demand. These areas range from ecology, life sciences, meteorology, health care, social, and economic sciences, mathematics and computer science to energy, food, and transport. Interdisciplinary approaches deliver huge amounts of data to create reliable future scenarios. They should provide a comprehensive understanding of the problem and possible measures at all levels. All models show significant geographical differences and illustrate the enormous burden on many developing countries. However, there is no in-depth analysis evaluating global research efforts on climate change including climate change-specific parameters, that provides a comprehensive picture with specific geographical and chronological patterns of scientific publications and the resulting needs and requirements for scientific action. Therefore, the present study focuses on the evaluation of the global and national publication output on climatic change to depict structures and international developments using bibliometric analyses. Metadata analysis allows a comprehensive assessment of the global scientific landscape because all countries are vulnerable to the impacts of climate change to varying degrees because of their natural and socioeconomic conditions.

Building on other bibliometric studies [2, 35], which also show the publication output of countries in the field of climate change, this analysis interprets global scientific output using country-specific indices relevant to climate change to present the world map accordingly [9, 13, 33]. The resulting implications help to answer the question of whether international research and networking on climate change meet global requirements and necessities given the current and predicted impacts on all regions and all areas of life. Thus, the interpretation of the results can enable decision makers, funders, scientists, and other stakeholders to develop concepts for future research based on carefully evaluated metadata.

## Methods

### Methodological platform

A representative and qualitative database has been built up, providing comprehensive metadata on the past and present scientific landscape of climate change research, its incentives, its benchmarks, and its challenges and requirements. The applied method is integrated into the bibliometric platform *New Quality and Quantity Indices in Science* (NewQIS), which was initiated in 2009 to provide in-depth data of the publication output on a variety of life science and biomedical topics [14, 17]. The approach combines the application of publication and contextual factors with state-of-the-art visualization techniques. The Core Collection Indices of Web of Science (WoS), which represent one of the most important scientific literature databases, are used as data sources. In addition, WoS provides citation parameters for advanced data interpretation and quality assessment via the *Journal Citation Report* (JCR) and the *Journal Impact Factor* (JIF).

### Search strategy, data acquisition, and correction

**Fig. 1:**
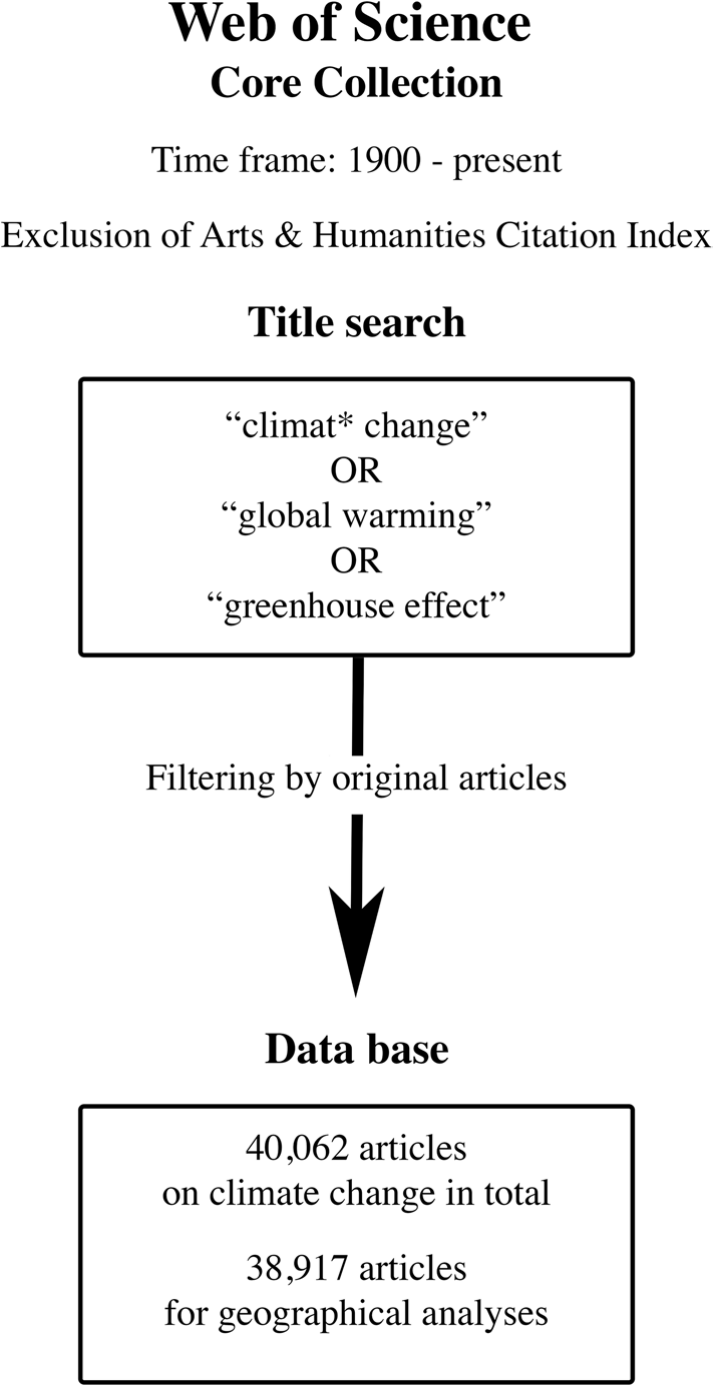
Procedure for generating the analysis database

The quality of the database depends on the appropriateness of the search strategy applied. The search term must involve all important synonyms. For this study the terms: “climat* change”, “global warming”, and “greenhouse effect” were applied. The asterisk acts as a wild card and was used to search for terms with different endings. To retrieve only the original research publications, only data from the publication type “Articles” was downloaded. The Art and Humanity entries were excluded. No limitation of the evaluation period was made so that all articles from 1900 to 2020 were included in the analysis (Fig. 1).

The aim was to decimate thematically incorrect entries and maximize correct ones. The risk of an unrepresentative database has been reduced by searching in the title of the manuscripts, even if e-data resulting from the search strategy cannot include all indexed articles. The metadata, sorted by various keyed information, was downloaded and saved as an MS-Access database. To unify different designations of data, e.g., the names of authors and their institutional affiliation, a standardization had to be carried out with the help of a specially developed application. For the standardization of institutions, a quantity of at least 200 articles in a regional context must be achieved. A threshold of at least 20 articles on climate change was set for authors. By applying those thresholds, it was possible to completely adjust all entries for institutions and authors above this value. Also, the names of the assigned subject areas had to be adapted and standardized due to missing spaces or typing errors. In doing so, all entries could be corrected without using a threshold value.

### Analysis parameters

The resulting database consists of a large number of bibliometric parameters. The research topics were clustered based on the keywords that occur at least 650 times (threshold) using the application VOSviewer [44].

Chronological analyses were carried out to evaluate the development of research (number of articles), research incentives (number of citations). In addition, geographical analyses were conducted to identify the main actors (countries with the most cited publications, most publishing institutions), and their international networking. The average citation rate of the countries is calculated by dividing the number of citations received by the number of the publication on climate change.

However, the evaluation of the absolute numbers does not allow an assessment of the development of publication shares and the current distribution of countries’ research output on climate change issues. Therefore, the evaluation period of the last 30 years was divided into 5-year intervals for further analysis, and the ten most publishing countries were analyzed.

By linking socioeconomic characteristics and citation parameters, important additional statements on country-specific publication activities on climate change can be made.

The country-specific number of articles was put in relation to (1) Demography: total population in million inhabitants (*R*_POP_) [39], (2) Socioeconomic status: gross domestic product (GDP) in billion US-Dollars (*R*_GDP_) [38], and (3) Research investment parameters: number of researchers in FTE (full-time equivalents) [40], expenditures on research and development (R&D) (personnel in FTE) [40], and gross expenditures for R&D (GERD) in PPP$ (purchasing power parity in US-Dollars) [40]. For all ratios, a minimum threshold of at least 30 articles was applied to avoid distortions due to extreme values.

For a more specific assessment of the national research contribution, it seems appropriate to include relevant country-specific indicators related to climate change. For this purpose, we select representative indices to put them in relation to the research output of the countries. CO_2_ emissions represent the responsibility of countries, the Global Climate Risk Index acts as a composite value for the different types of damage that countries can expect, the rise in sea level as a measure of the enormous global environmental impact, and the readiness and vulnerability index for the different circumstances of the individual countries under which climate change will occur.

1. Carbon dioxide (CO_2_) emission in tons per year [33]: The integration of the CO_2_ emissions of the countries was done by calculating the relation of the number of articles to CO_2_ emissions in billions of tons (threshold = 300).
2. The Global Climate Risk Index (CRI): The CRI was launched by German Watch and published in its 15th edition 2020. It assesses the extent to which countries have been suffering from weather incidences [9]. The CRI provides data for the last 20 years as an average value and also for individual years. The existing data on the weather vulnerability, measured in fatalities per country, and losses in US dollars could indicate the expected increase in extreme events due to climate change and help to mitigate the impacts.
3. Sea-level rise: For the analysis of the number of people living on vulnerable land due to prognosticated sea level rise [19], we have taken the values of the average number of fatalities per 100,000 inhabitants from 1999 to 2018 as reference quantity. To estimate the resulting sea-level rise, the US National Aeronautics and Space Administration (NASA) created the *digital elevation model* (DEM) SRTM (*Shuttle Radar Topography Mission*). The here utilized CoastalDEM is a development based on the neural networks to reduce SRTM errors resulting from its limitation with respect to terrain elevations (important for densely populated areas) by regression analysis [19]. There are several prospective scenarios, based on the 5th IPCC report [15], which are based on the Representative Concentration Pathways (RCP) models 2.6, 4.5, and 8.5 leading to different degrees of global temperature rise. These scenarios presuppose different greenhouse gas concentrations. RCP 8.5 would lead to a rise of 4 °C, while RCP 4.5 would lead to a rise of 2.6 °C, and the target limit of 2 °C set by the Paris Agreement can be realized by the RCP 2.6 scenario—always as compared to pre-industrial times [10]. In addition, the Sea Level Rise Modell K17 is a nonprobabilistic projection that incorporates physical models of ice sheet dynamics [18]. Furthermore, the applied model data refer to the forecast for the year 2100 and include the local 1-year coastal flood return level [19]. For our analysis, we chose the K17 model, CoastalDEM, RCP 4.5 for the year 2100.
4. Readiness and vulnerability index: To assess the differences between the individual countries, the *Notre Dame Global Adaptation Initiative* (ND-GAIN) developed a country index that provides data on countries’ vulnerability to climate disruption and their readiness to improve resilience by “leverage of private and public sector investments”. The index combines 74 variables to define the ranking for 192 countries [27].

### Visualization of results

The results of the keyword cluster analysis were presented using the VOSviewer software developed by van Eck and Waltman [44]. The occurrence of keywords was visualized by a network of nodes and connecting lines representing the different colored clusters and their combinations.

The geographical findings of this study were partially visualized by the creation of anamorphic cartograms using Gastner and Newman’s method of density equalizing map projections (DEMP) [12]. Methodically, these DEMPs reduce or enlarge the country sizes according to the value of the evaluation parameter, following the physical principle of density compensation by diffusion balance in each country. To maintain the basic structure of the world map, mean values are calculated and assigned to oceans and Antarctica. With an ArcGIS tool (mapping software for geographic information systems), which is based on the algorithms of the DEMP method, geographic data can be visualized by generating distorted maps. The DEMPs generated in this way allow a quick visual acquisition of the extensive data and concentration on the essential.

### Methodological limitations and strengths

Although being a sophisticated and widely applied method, some limiting points need to be recognized and discussed.

The quality and representativeness of the retrieved metadata depend on the one hand on the technical and bibliographic conditions of the source database and on the other hand on the care taken in generating the search strategy. In this case, WoS was used as a data source. It should be noted that WoS is English biased, as most of the indexed journals are English-language journals. Furthermore, the citation number given is prone to various errors, e.g., incorrect citation behavior or self-citation, so that its significance for the quality of research needs to be discussed. Although the strategy of searching only in the title of the publications resulted in a reduced data quantity, this is justified by the higher representativeness of the data sets. The additional search in the abstracts and keywords would lead to the inclusion of a large number of false entries that would not provide valid figures. Therefore, choosing a title search strategy allows the creation of a valid, albeit not all-encompassing, database.

Some data records had to be corrected manually, e.g., institutions and subject areas. Although the unification of the subject areas in the overall database could be carried out exactly, the merging of different labeled affiliations belonging together was not 100% possible. Therefore, a threshold has been applied in a geographical approach, so that only those geographical entity, e.g., cities, with at least 200 articles on climate change were subject to in-depth corrections. However, the exact number of publishing institutions could not be determined.

The visualization of the results utilizing DEMPs is limited by the physical principles of the technique so that some small island countries could not be represented in the respective figures.

## Results

All the results are based on the evaluation database, which consists of a total of 40,062 articles on climate change identified and extracted from WoS.

### Research focal points

**Fig. 2:**
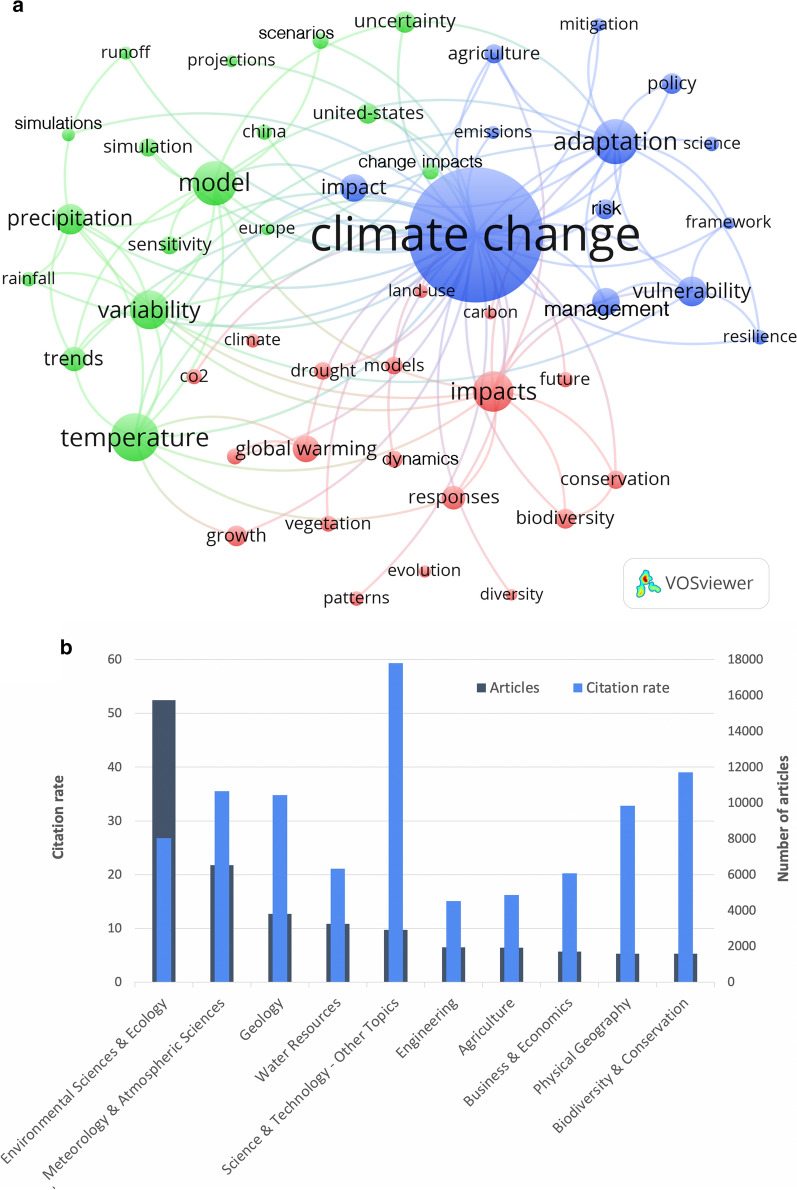
Research foci. **a** Clusters of author’s keywords with at least 650 occurrences. Red: environmental and ecological issues, green: modeling and simulation issues, blue: social and management issues. **b** Most assigned subject areas according to Web of Science categories with number of articles and average citation rate (number of citations / number of articles)

In total, 45 keywords from three main clusters could be identified (Fig. 2a). First, articles relating to environmental and ecological issues can be grouped together, with "impacts" being the most commonly used term in the cluster. Secondly, all articles dealing with modeling and simulation can be grouped. In this second keyword cluster, the terms “temperature”, “model”, and “variability” appeared most frequently. Thirdly, all articles on social, political, and management issues can be grouped in one cluster. The umbrella term “climate change” was assigned to this group and is the most frequently used keyword in the analysis. In addition, the terms “adaption” and “vulnerability” have been used most frequently in the third keyword cluster.

The main subject areas (WoS research areas) are shown in figure Fig. 2b with the numbers of articles (*n*) assigned to them and their average citation rates. By far the most assigned subject area was *Environmental Science and Ecology* (*n* = 15,741). *Meteorology and Atmospheric Science* (*n* = 6522) followed with less than half of assigned articles. Ranks 3 to 5 were occupied by *Geology* (*n* = 3806), *Water Resources* (*n* = 3247), and *Science and Technology—Other Topics* (*n* = 2916). Apart from ecological issues, the most frequently assigned subject areas (*Business and Economics: n* = *1710*, *Government and Law: n* = *1493*, *Public Administration: n* = *936*) focus on economics and political issues, which represent the blue cluster in Fig. 1a. In principle, the articles are distributed over the three main subject areas clusters that distinguish between scenario modeling, risk analysis, and mitigation, respectively, adaption measurements. From these results, the main foci of climate change research can be identified. In summary, articles on modeling and simulation of scenarios for consequences of climate change under different conditions have been developed resulting in ecological and socioeconomic impacts, which in turn form the basis for mitigation and adaption measures on climate change.

It is noteworthy concerning the average citation rate (cr) of the research areas that the highest rates reached the areas *Science and Technology—Other Topics* (cr = 59.37), *Biodiversity and Conservation* (cr = 39.01), *Geography* (cr = 38.85), and *Meteorology and Atmospheric Science* (cr = 35.54), while the most assigned area *Environmental Science and Ecology* achieved an average of only cr = 26.80. Among the ten most frequently assigned subject areas, *Public Administration* (cr = 13.49) and *Government and Law* ranked last (cr = 10.69).

### Evolution of publication output over time

**Fig. 3:**
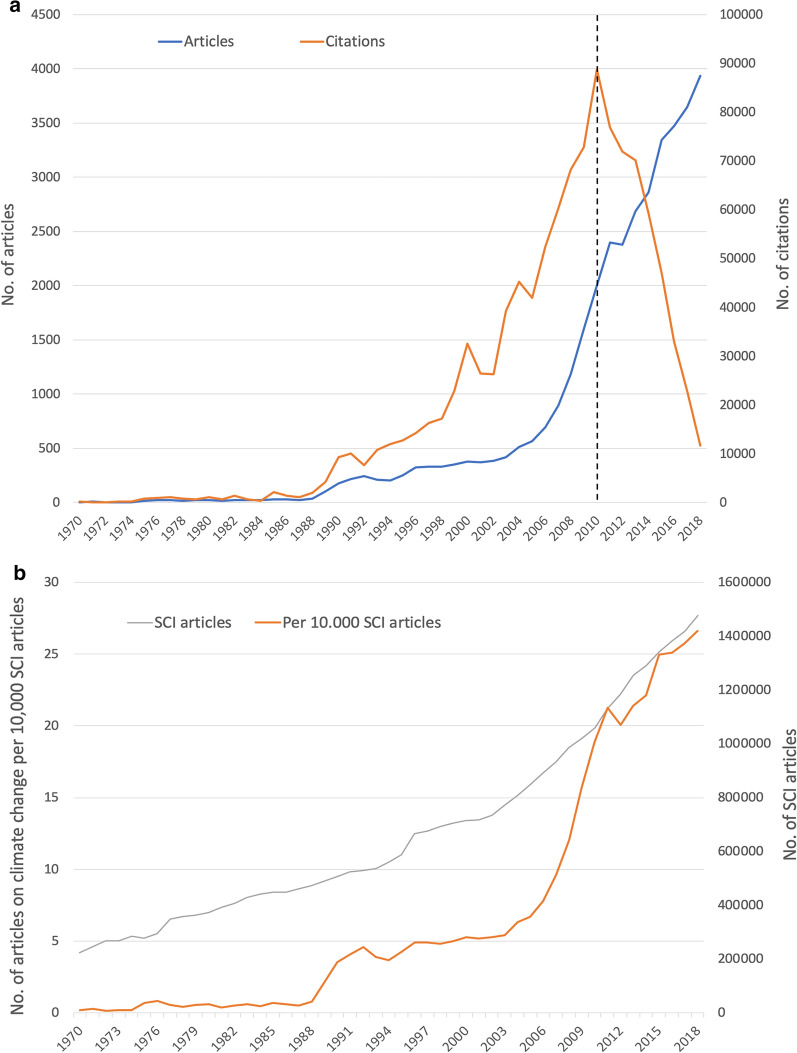
Chronological development of articles on climate change from 1970 to 2018. **a** Number of articles on climate change and their citations. Dashed line: Cited Half-Life. **b** Number of all indexed SCI articles (Science Citation Index of Web of Science) and number of articles on climate change per 10,000 SCI articles

The vast majority of articles on climate change (92.17%) has been published since the year 2000 (*n* = 36,925) (Fig. 3). However, the first publication that meets the search criteria was published as early as 1910. Annual publication numbers remained in single digits until the mid-1970s. Only at the end of the 1980s, the numbers reach yearly amounts above *n* = 100. A steep increase in research activity can be observed from 2003 onwards when the trend followed an exponential course, which reached a small peak in 2011 and is still rising exponentially until today (Fig. 3a). This development can be illustrated even more clearly by looking at the numbers in relation to the absolute number of articles indexed in the *Science Citation Index* (SCI) (Fig. 2b). The gradual increase in research interest is also reflected in the steep relative increases in these years, calculated with the annual number of articles on climate change per 10,000 articles listed in the *Science Citation Index* (SCI) (Fig. 3b). Until 1988 and between 1992 and 2003, the upward trend of climate change research is similar to that for all articles indexed in the SCI.

Analog to the development of the number of articles, the number of citations (c) also increased significantly since 1988, with peaks in the years 1991 (*c* = 10,106), 2000 (*c* = 32,612), 2004 (*c* = 45,177), and the preliminary maximum of *c* = 88,747 in 2010. Afterward, the citation numbers dropped again significantly. This is because little time has elapsed since the articles were published to generate citations. This effect is known as Cited Half-Life (CHL) and refers to a period of about eight years for the life sciences, which is needed for the articles to reach half of the total number of citations (Fig. 3a) [21].

Among the ten most frequently cited articles in the database, 80% stem can from the USA (*n* = 8) and 20% from the UK (*n* = 2). All of those ten articles were published after 2000, and mainly in the renowned journals *Nature* (*n* = 5) and *Science* (*n* = 2) (Tables 1, 2). The publication years 2000, 2003, and 2010 can be logically associated with the research increase shown in Fig. 2a.

**Table 1:** The most cited original articles on climate change until November 2019, * country of origin of first author

Author	Country*	Year	Citations	Title	Journal
Parmesan et al	USA	2003	5305	A globally coherent fingerprint of climate change impacts across natural systems	Nature
Thomas et al	UK	2004	3770	Extinction risk from climate change	Nature
Moss et al	USA	2010	2870	The next generation of scenarios for climate change research and assessment	Nature
Allen et al	USA	2010	2819	A global overview of drought and heat-induced tree mortality reveals emerging climate change risks for forests	Forest Ecology and Management
Root et al	USA	2003	2579	Fingerprints of global warming on wild animals and plants	Nature
Lal	USA	2004	2534	Soil carbon sequestration impacts on global climate change and food security	Science
Cox et al	UK	2000	2222	Acceleration of global warming due to carbon-cycle feedbacks in a coupled climate model	Nature
Held, Soden	USA	2006	2141	Robust responses of the hydrological cycle to global warming	Journal of Climate
Vorosmarty	USA	2000	2075	Global water resources: Vulnerability from climate change and population growth	Science
Meehl et al	USA	2007	1887	The WCRP CMIP3 multi model dataset—a new era in climate change research	Bulletin—American Meteorological Society

**Table 2:** The most publishing institutions on climate change

Institution	Country	Articles	Citations	Citation rate
Chinese Academy of Science	China	1333	28,383	21.29
University of London	UK	680	29,365	43.18
USDA	USA	588	23,394	39.79
University of Oxford	UK	452	24,508	54.22
Wageningen University	Netherlands	442	18,700	42.31
University of Washington	USA	426	24,440	57.37
US Geological Survey	USA	381	17,177	45.08
National Center for Atmospheric Research	USA	380	37,599	98.94
Columbia University	USA	380	28,560	75.16
University of East Anglia	UK	366	32,634	89.16
University of Amsterdam	Netherlands	352	13,427	38.14
University of Colorado	USA	333	14,473	43.46
Potsdam Institute of Climate Impact Research	Germany	333	20,383	61.21
University of California Berkeley	USA	332	21,697	65.35
ETH Zurich	Switzerland	318	14,984	47.12

### Leading institutions

The 15 most publishing institutions on climate change are located exclusively in the northern hemisphere (Table 2). Almost half of the most publishing institutions are US-American (7 institutions), 3 others are British, 2 are Dutch, and 1 institution is located in China, Switzerland, and Germany respectively. The Chinese Academy of Science (CAS) was the most publishing institution on climate change with *n* = 1333 articles, followed by the University of London (*n* = 680), which published only half the amount. The US Department of Agriculture (USDA) followed with *n* = 588 articles. In 4th place was the British University of Oxford (*n* = 452), followed by the Dutch Wageningen University (*n* = 442) and the US University of Washington (*n* = 426). When looking at the average citation rate of the most publishing institutions, the order is different. With the highest value of almost 100, the US National Center for Atmospheric Research (cr = 98.94) led the ranking, followed by the British University of East Anglia (cr = 89.16), and the US Columbia University (cr = 75.16). The articles of the CAS ranked last among the leading 15 institutions (cr = 21.29).

### Global landscape of publication output

Not all articles out of the entire database could be assigned to a country of origin due to missing metadata before 1973. Coming from 186 countries or autonomous regions, *n* = 38,917 articles could thus be included in the database and analyzed in terms of geographical parameters.

**Fig. 4:**
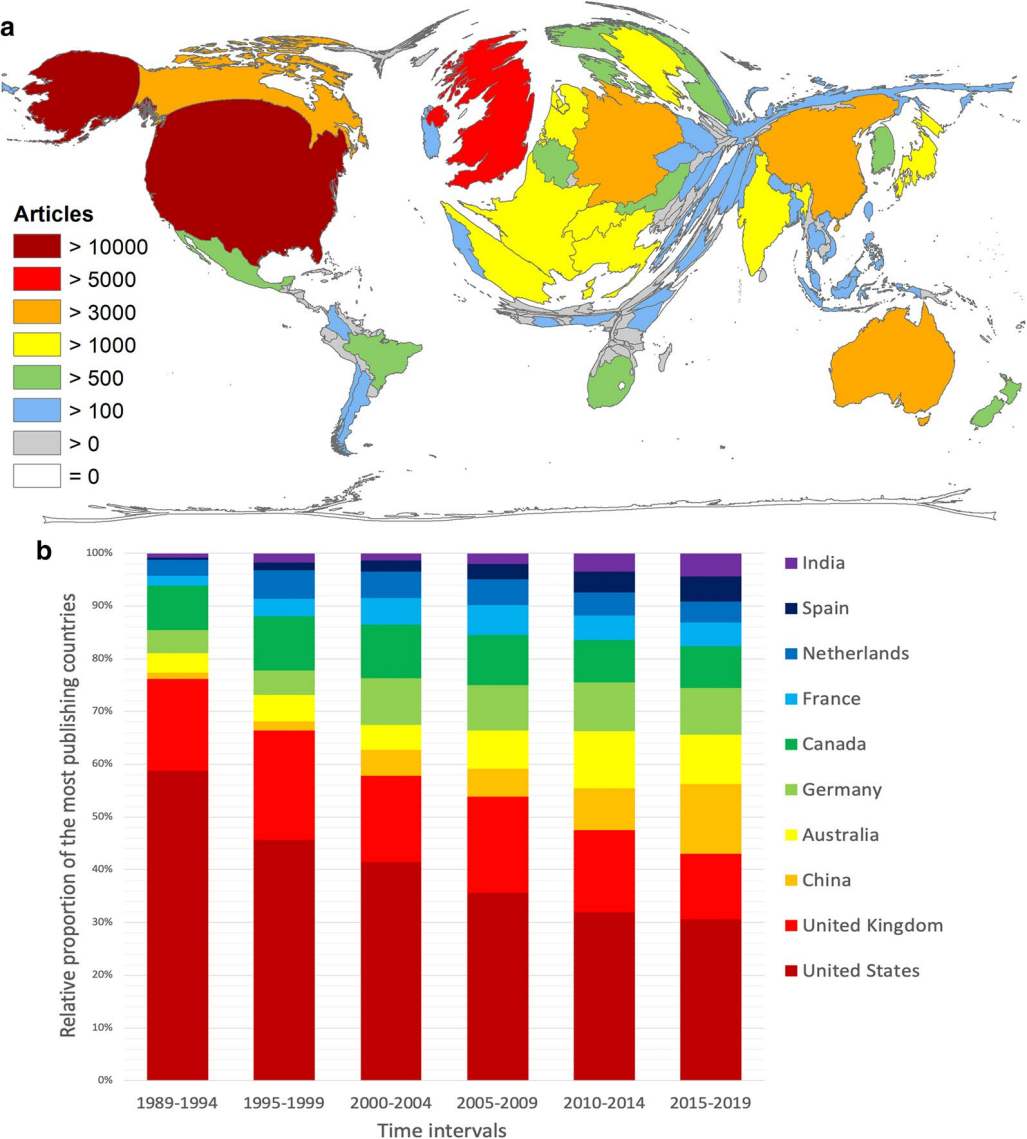
The most publishing countries. **a** Density equalizing map projection of the number of articles. **b** Relative share of the most publishing countries in 5-year intervals from 1998 to 2019

The most publishing country was the USA with *n* = 12,637 articles on climate change, followed by the United Kingdom (UK) with less than half as many articles (*n* = 5524). China was placed 3^rd^ with n = 3508, followed by Australia (*n* = 3349), Germany (*n* = 3238, and Canada (*n* = 3126) (Fig. 4a).

Looking at the share of the most publishing countries in 5-year intervals (Fig. 4b), the USA conducted more than 50% of the research on climate change in the first evaluation interval from 1989 to 1994. In the last interval from 2015 to 2019, the share of US articles decreased to 30%, whereas the absolute numbers increased almost tenfold. The relative share of the UK fell also from 20.64% to 12.42% between 1995 and 2019, during which time it lost its second rank to China that contributed an increasing share from 1.23% to 13.27% throughout the whole evaluation period. The share of Australian, German, Spanish, and Indian articles also increased slightly over time, while the shares of Canadian, French, and Netherlandic articles remained more or less the same.

**Fig. 5:**
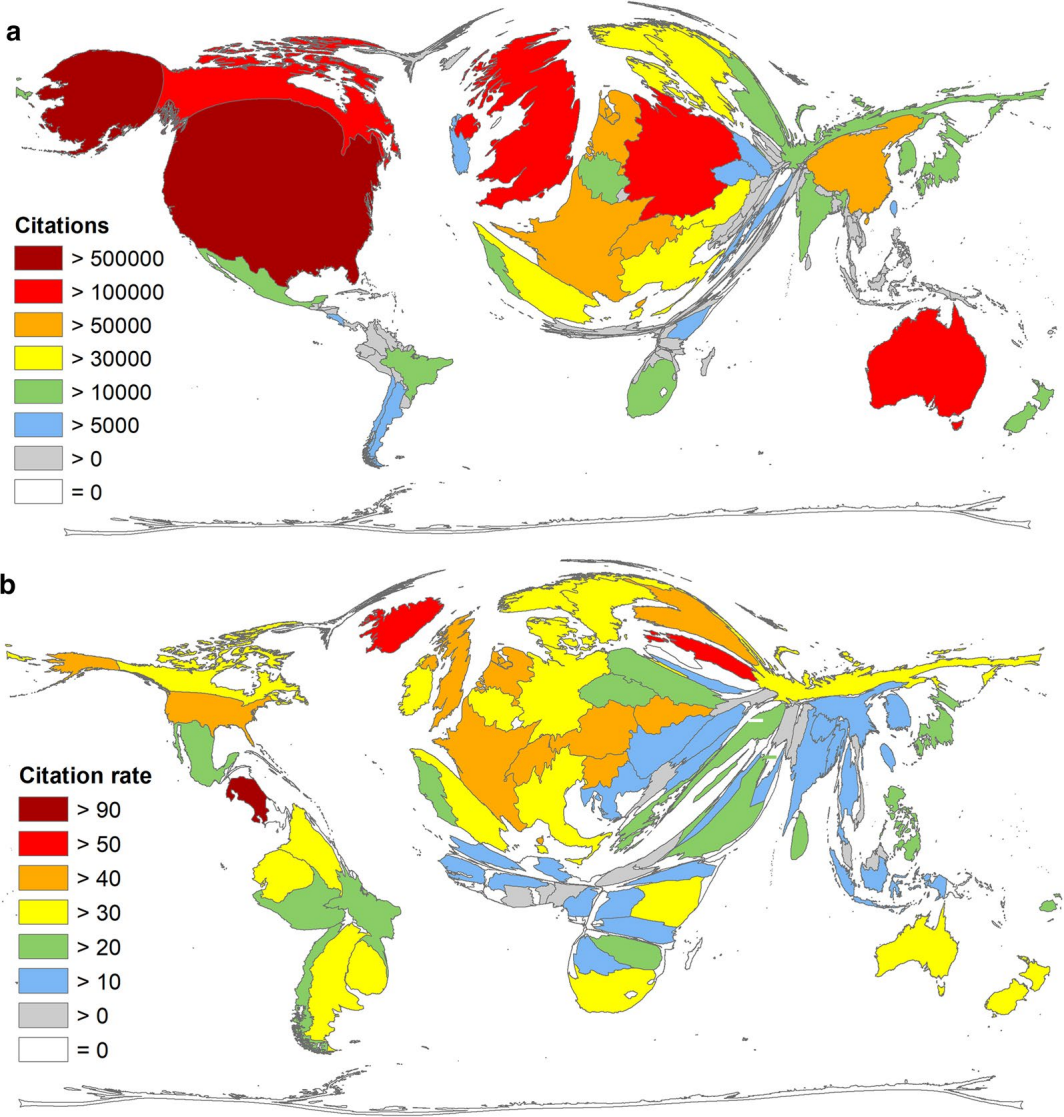
Citation-specific parameters for articles on climate change. **a** Number of citations per country. **b** Articles/Citation rate of articles on climate change per country (threshold 30 articles)

The distribution of the number of citations follows a similar pattern with the exception of China, which here falls to rank 8 (*c* = 66,844). The USA received by far the most citations (*c* = 513,888), followed by the UK (*c* = 243,261), Australia (*c* = 108,054), Canada (c = 107,713), and Germany (*c* = 107,335) (Fig. 5a).

When evaluating the average citation rate (cr) per country with more than 30 articles on climate change (threshold), Costa Rica was in first place (cr = 93.89, *n* = 67), followed by Estonia (cr = 55, *n* = 66), Iceland (cr = 50.15, *n* = 47), Austria (cr = 46.77, *n* = 668), and Switzerland (cr = 45.94, n = 1126). The UK ranked 16th (cr = 44.04), the USA 21st (cr = 40.66), Canada 35th (cr = 34.46), Germany 40th (cr = 33.15), and Australia 41st (cr = 32.26) (Fig. 5b).

### Inclusion of socioeconomic parameters

The analysis of socioeconomic parameters of the publishing countries on climate change showed a divergent ranking.

**Fig. 6:**
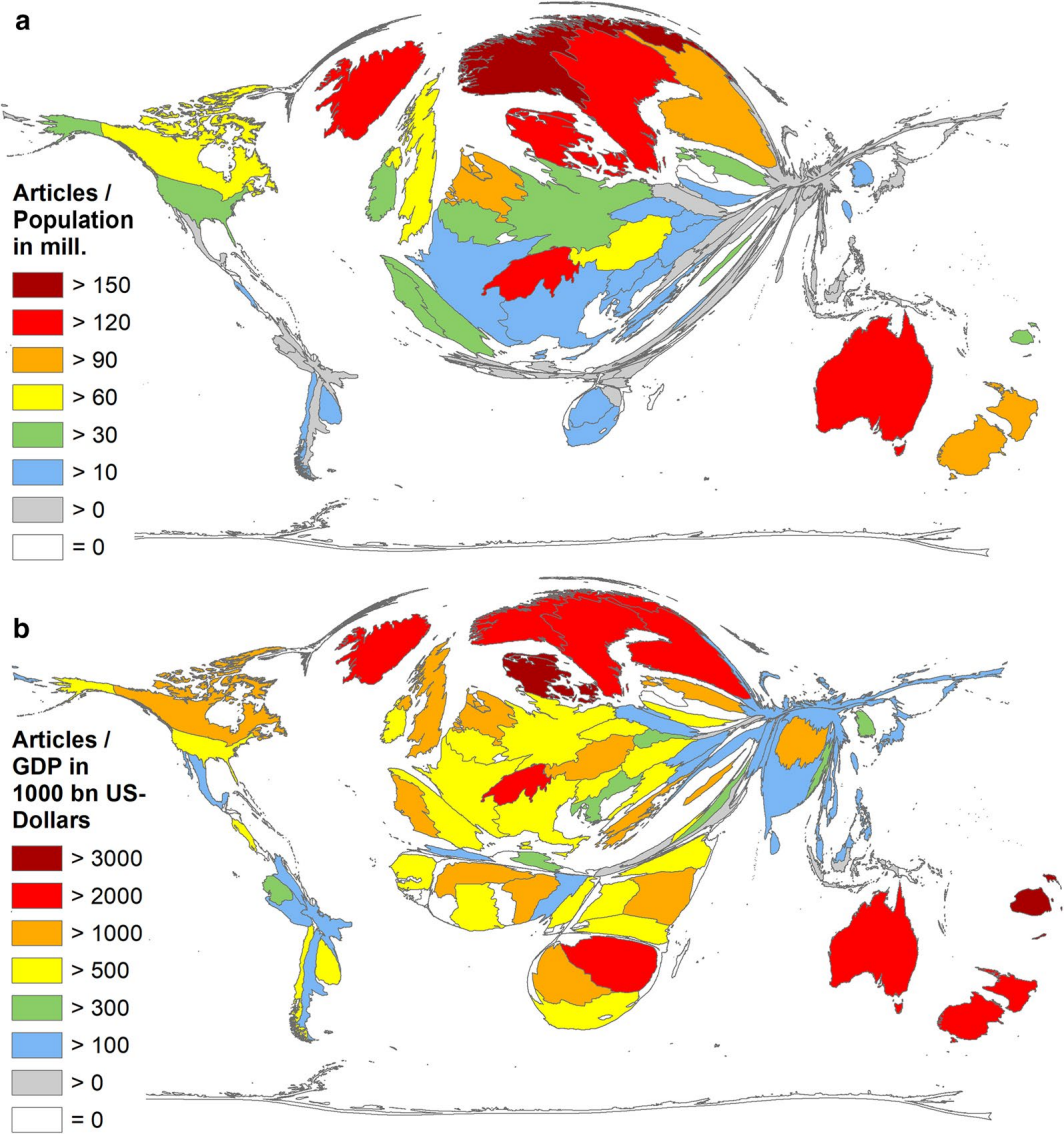
Ratio of socio-economic parameters (threshold 30 articles). **a** Country-specific ratios of the number of articles on climate change and the countries’ population size in million inhabitants [39]. B) Country-specific ratios of the number of articles on climate change and the Gross Domestic Product (GDP) in 1000 billion US-Dollars [38]

In terms of the inclusion of the countries’ population size [39] (number of articles/population in million inhabitants = *R*_POP_) the following order emerged: Norway (*R*_POP_ = 174.16), Australia (*R*_POP_ = 145.65), Denmark (*R*_POP_ = 142.12), Iceland (*R*_POP_ = 139.93), Switzerland (*R*_POP_ = 137.66). The most publishing countries were ranked lower: the USA ranked 20th (*R*_POP_ = 39.00), the UK ranked 13th (*R*_POP_ = 85.73), China ranked 67th (*R*_POP_ = 2.55), and Germany ranked 18th (*R*_POP_ = 40.11) (Fig. 6a).

In terms of the economic status, the South Pacific island state Fiji led the range of countries with more than 30 articles on climate change (threshold) with a ratio of the numbers of articles and the GDP in billion US-Dollars [38] (*R*_GDP_) with *R*_GDP_ = 6329.11, followed by Denmark (*R*_GDP_ = 3002.26), New Zealand (*R*_GDP_ = 2991.99), Iceland (*R*_GDP_ = 2910.22), and Australia (*R*_GDP_ = 2816.65) (Fig. 6b). It was also surprising that the African country Zimbabwe was placed among the top ten countries and reached 7th place (*R*_GDP_ = 2541.48). In terms of socioeconomic analysis, other developing countries such as Nepal and some African countries (Kenya, Benin) achieved also ranks among the leading 20 countries.

Besides Australia, the UK reached the second highest ratio of the most publishing countries and ranked 11th, Canada ranked 13th, Germany 30th. The USA was only in 37th position.

The inclusion of science-related parameters [40]. listed New Zealand first (Table 3) with *R*_GERD_ (number of articles/gross expenditure for research and development in current PPP (purchasing power parity) US dollars) = 244.34. Australia ranked 2nd (*R*_GERD_ = 157.98), followed by Norway (*R*_GERD_ = 133.77), South Africa (*R*_GERD_ = 120.36), and UK (*R*_GERD_ = 115.54). Germany only achieved rank 22nd (*R*_GERD_ = 25.47), and the USA ranked 23rd (*R*_GERD_ = 23.26).

New Zealand published also the highest number of articles per researcher (full-time equivalent FTE/1000) with *R*_RES_ = 27.92, followed by Norway, South Africa, and Switzerland. Here the USA ranked 16th (*R*_RES_ = 9.22) and Germany 19th (*R*_RES_ = 7.83). Unfortunately, the data for Australia was not available.

**Table 3:** Number of articles on climate change (threshold 300 articles) related to research specific parameters per country ranked by *R*_GERD_, GERD in bn PPP$ = gross expenditures for research and development in billion in US dollars (PPP = purchasing power parity), FTE = full-time equivalent, *R*_GERD_ = number of articles per GERD in current bn PPP$, and *R*_RES_ = number of articles per Researcher FTE/1000

Rank *R*_GERD_	Country	Articles	GERD in bn PPP$	Researcher FTE/1000	*R*_GERD_	R_RES_	Rank *R*_RES_
1	New Zealand	523	2.14	18.70	244.34	27.97	1
2	Australia	3349	21.20	n/a	157.98	n/a	n/a
3	Norway	917	6.86	34.37	133.77	26.68	2
4	South Africa	733	6.09	27.66	120.36	26.50	3
5	UK	5524	47.81	289.67	115.54	19.07	6
6	Canada	3126	27.18	155.13	115.01	20.15	5
7	Iran	339	3.32	51.96	102.19	6.52	20
8	Portugal	429	4.33	44.32	99.13	9.68	14
9	Finland	637	6.83	37.05	93.28	17.19	9
10	Greece	316	3.40	35.19	93.02	8.98	17
11	Netherlands	1595	18.01	85.30	88.58	18.70	7
12	Denmark	795	9.20	45.28	86.46	17.56	8
13	Sweden	1215	16.74	75.25	72.57	16.15	11
14	Spain	1458	21.37	133.20	68.23	10.95	13
15	Switzerland	1126	17.79	43.74	63.30	25.74	4
16	Austria	668	14.58	44.93	45.81	14.87	12
17	Mexico	501	11.03	29.92	45.44	16.74	10
18	Italy	1300	32.47	136.20	40.03	9.54	15
19	Belgium	503	14.18	56.48	35.48	8.91	18
20	France	1724	62.95	288.58	27.39	5.97	21
21	India	1300	49.75	282.99	26.13	4.59	22
22	Germany	3238	127.11	413.54	25.47	7.83	19
23	USA	12,637	543.25	1371.29	23.26	9.22	16
24	Brazil	688	39.90	179.99	17.24	3.82	23
25	Turkey	305	20.58	111.89	14.82	2.73	24
26	Russia	488	42.27	410.62	11.55	1.19	28
27	China	3508	495.98	1740.44	7.07	2.02	25
28	South Korea	621	89.83	383.10	6.91	1.62	27
29	Japan	1106	175.84	676.29	6.29	1.64	26

Researcher FTE data for Australia was not available (n/a) [40]

### Inclusion of climate change indices

#### Carbon dioxide emission

The linkage of country-specific number of publications on climate change with the countries’ CO_2_ emission shown in Table 4 discloses Sweden as the leading country (*R*_CO2_ = 29.28), followed by Switzerland (*R*_CO2_ = 28.10), Denmark (*R*_CO2_ = 23.01), Norway (*R*_CO2_ = 20.47) and New Zealand (*R*_CO2_ = 14.52). In this analysis, the most publishing countries fell sharply behind. UK ranked 6^th^ (*R*_CO2_ = 14.36), Germany 17th (*R*_CO2_ = 4.05), and the USA 19th (*R*_CO2_ = 14.52).

**Table 4:** Number of articles on climate change related to the countries’ CO_2_ emission in billion tons (threshold 300 articles), countries are ranked by *R*_CO2_ = number of articles/CO_2_ emission in billion tons [40]

Country	Articles	CO_2_ in bn t	*R*_CO2_
Sweden	1215	41.50	29.28
Switzerland	1126	40.07	28.10
Denmark	795	34.55	23.01
Norway	917	44.79	20.47
New Zealand	523	36.01	14.52
United Kingdom	5524	384.71	14.36
Finland	637	45.96	13.86
Netherlands	1595	164.05	9.72
Austria	668	69.94	9.55
Australia	3349	413.09	8.11
Portugal	429	54.86	7.82
Canada	3126	572.78	5.46
Spain	1458	281.42	5.18
Belgium	503	100.12	5.02
France	1724	356.30	4.84
Greece	316	76.00	4.16
Germany	3238	799.37	4.05
Italy	1300	355.45	3.66
United States	12,637	5269.53	2.40
South Africa	733	456.33	1.61
Brazil	688	476.07	1.45
Mexico	501	490.29	1.02
South Korea	621	616.10	1.01
Japan	1106	1205.06	0.92
Turkey	305	447.90	0.68
India	1300	2466.77	0.53
Iran	339	672.31	0.50
China	3508	9838.75	0.36
Russia	488	1692.79	0.29

#### Global climate risk index

**Fig. 7:**
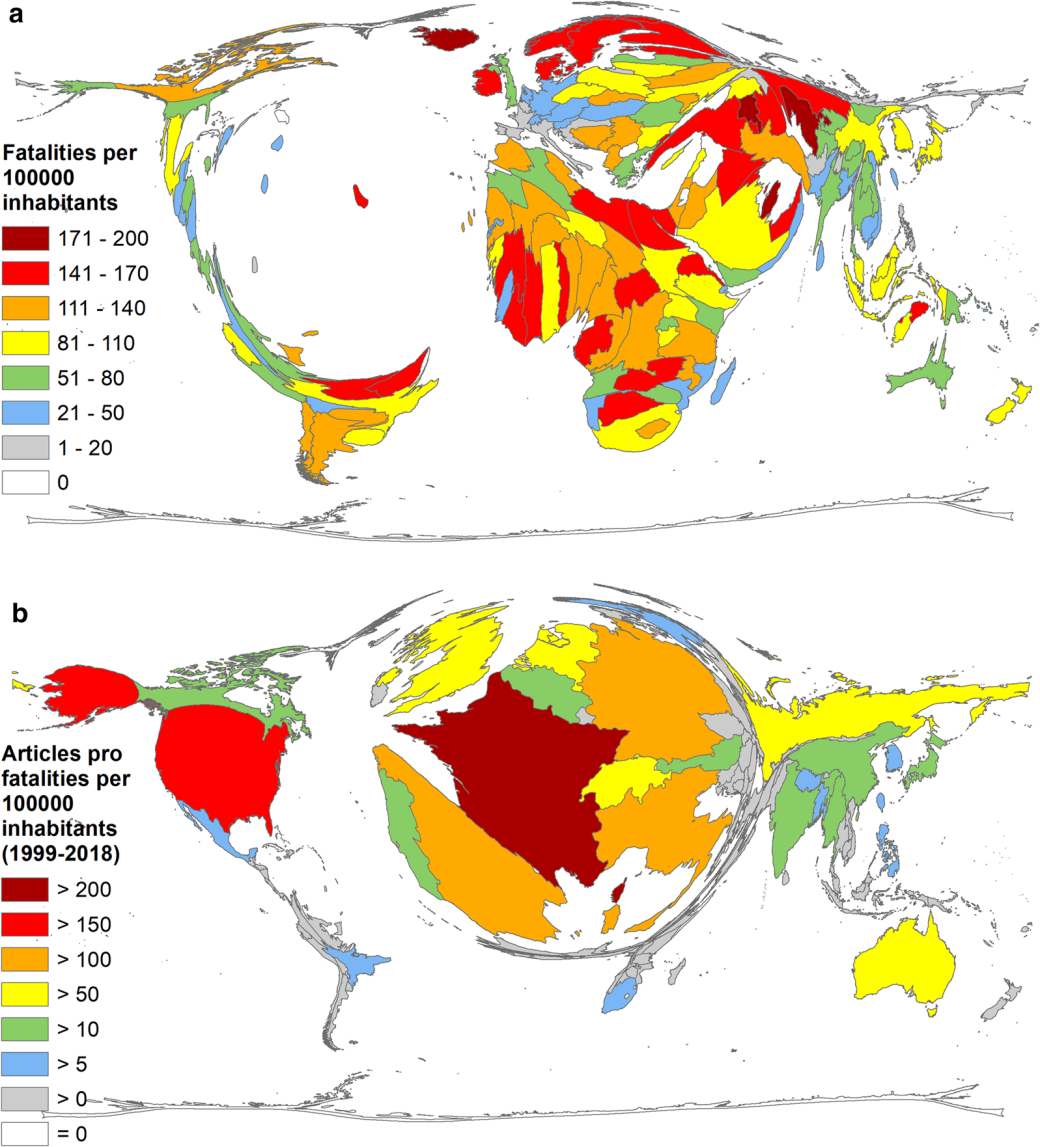
Global Climate Risk Index (1999–2018) [9]. **a** Average number of fatalities per 100,000 inhabitants. **b** Number of articles on climate change pro average number of fatalities per 100,000 inhabitants

For reasons of comparison, reference is made here to the results of the Global Climate Risk Index (CRI) [9]: The average ranking shows Puerto Rico, Myanmar, and Haiti as the most affected countries, while the assessment for 2018 ranked Japan, the Philippines, and Germany as the most affected countries [9]. The figures for the average number of deaths per 100,000 inhabitants from 1999 to 2018 as a reference point put some small island developing states (SIDS), such as St. Kitts and Nevis, Tuvalu, Kiribati, Seychelles, Marshall Islands, and the Maldives in the first place. Armenia, Iceland, Singapore, and Qatar also held leading positions. (Fig. 7a).

The linkage of the number of publications on climate change to the expected increase in extreme events due to climate change discloses France as the leading country (*R*_CRI_ = 215.50), followed by the USA (*R*_CRI_ = 162.01), Spain (*R*_CRI_ = 145.80), Italy (*R*_CRI_ = 144.44), Germany (*R*_CRI_ = 140.78), and UK (*R*_CRI_ = 215.01). Of the countries most affected by climate change, Myanmar ranked 17th (*R*_CRI_ = 12.00), followed by Japan on rank 18 (*R*_CRI_ = 11.89), and the Philippines on rank 20 (*R*_CRI_ = 8.19) (Fig. 7b). There was no correlation between the number of articles and the average number of fatalities per 100,000 inhabitants on average.

#### Sea-level rise

**Fig. 8:**
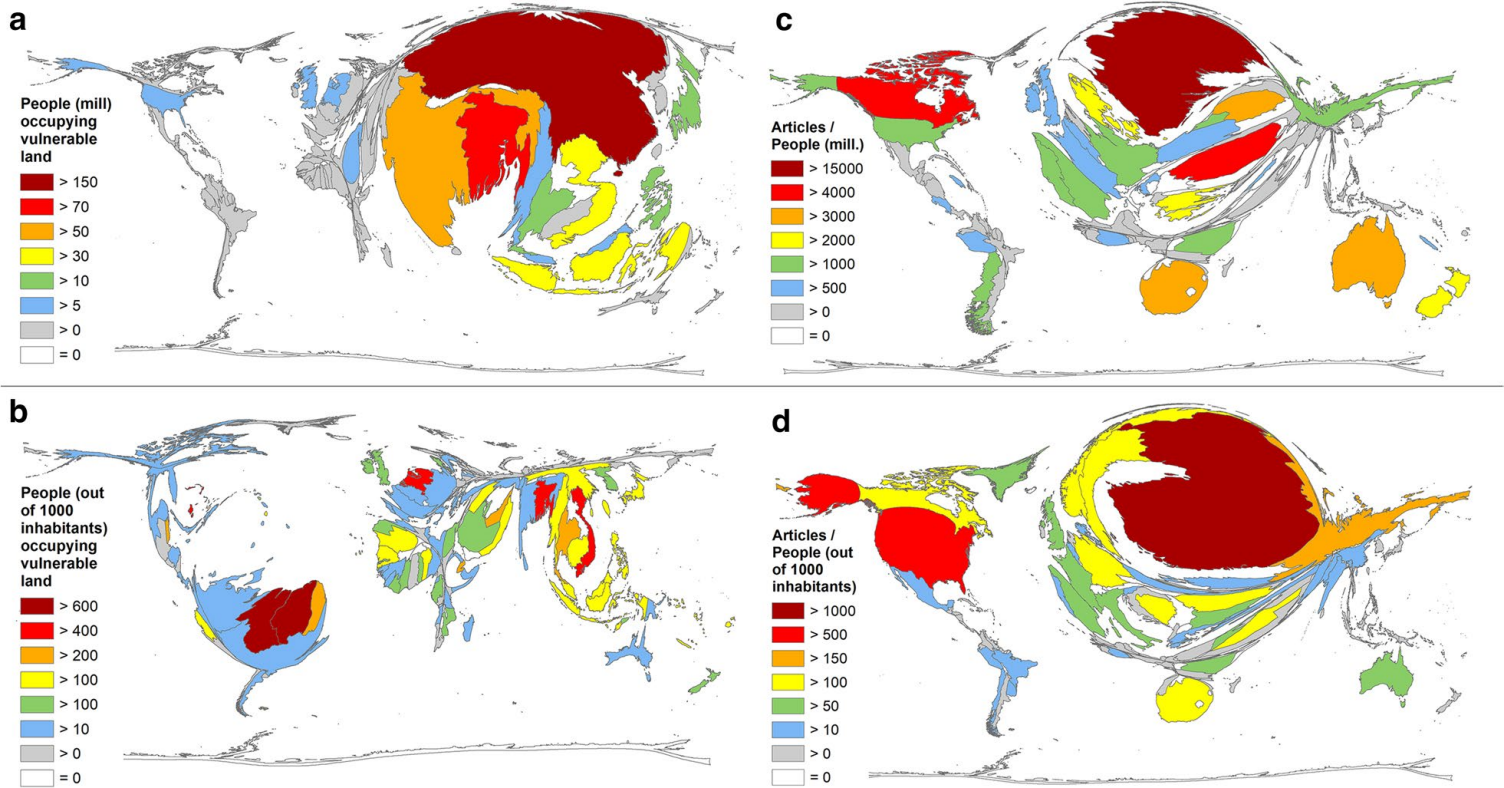
Estimated number of people exposed to vulnerable land in 2100 (CoastalDEM scenario: Sea Level Rise Modell K17, RCP 4.5, 95 percentile) [19]. **a** Number of people living on vulnerable land in mill. **b** Relative number of people (per 1000 inhabitants) living on vulnerable land. **c** Relation of the number of articles on climate change and the number of people living on vulnerable land in mill. High values of SIDS (Small Island Developing States) cannot be shown. The highest values have Maldives (87%), Marshall Island (85%), Tokelau (78%), Tuvalu (73%). **d** Relation of the number of articles on climate change and the relative number of people (per 1000 inhabitants) living on vulnerable land in mill

For reasons of comparison, reference is made here to the results of Kulp and Strauss [19]: According to their findings of the working group, China is by far the country with the highest number of people living on vulnerable land in million according to the CoastalDEM scenario (we here label it: *P*_vul_ = 151.6) (Fig. 8a). With *P*_vul_ = 73, Bangladesh’s population is the second most affected, followed by India (*P*_vul_ = 151.6), Vietnam, and Indonesia (*P*_vul_ = 151.6). In addition to these absolute figures, the working group of Kulp and Strauss [19] put the number of affected people in relation to the total population. This results in a different picture (Fig. 8b), with the small island states (Maldives, Marshall Islands, Tokelau, and Tuvalu) most affected, where more than 70% of the population will live on vulnerable land in 2100. In the South-American countries of Suriname and Guyana, more than 60% will live on vulnerable land, followed by Kiribati, Cayman Islands, and the Bahamas with more than 50% affected people. The Netherlands is the first European country in the ranking, where 55% of the inhabitants will be exposed to vulnerable land. The here determined most publishing countries on climate change, were following far behind: USA (2.3%), UK (9%), China (11%), Australia (4%), and Germany (2.5%) [19].

Here we have calculated the ratio of countries’ publication performance on climate change in relation to Kulp et al.’s absolute (*R*_absolute_) and relative figures (*R*_relative_) of Kulp and Strauss [19] (Fig. 7c, d). In terms of the relation of articles on climate change to the absolute numbers, Sweden was leading (*R*_absolute_ = 15,187), followed by Canada (*R*_absolute_ = 4597), Romania (*R*_absolute_ = 4366), Australia (*R*_absolute_ = 3940), South Africa (*R*_absolute_ = 3054), and Lithuania (*R*_absolute_ = 3050). The most publishing countries ranked as follows: The USA ranked 11th (*R*_absolute_ = 1805), Germany 14th (*R*_absolute_ = 1619), and UK 17th (*R*_absolute_ = 986), while China followed far behind on rank 88 (*R*_absolute_ = 23).

The analysis of the relative ratios led to the following ranking: Finland (*R*_relative_ = 2123), USA (*R*_relative_ = 549), Russia (*R*_relative_ = 157), South Africa (*R*_relative_ = 150), Canada (*R*_relative_ = 149). In terms of most publishing countries, Germany was ranked 8th (*R*_relative_ = 130), UK 18th (*R*_relative_ = 61), and China 25th (*R*_relative_ = 32).

A significant correlation could be shown between the absolute numbers of people living on vulnerable land and the number of articles (*p* < 0.001), while the relative numbers did not correlate with the number of articles (*p* < 0.53).

#### Vulnerability and readiness

**Fig. 9:**
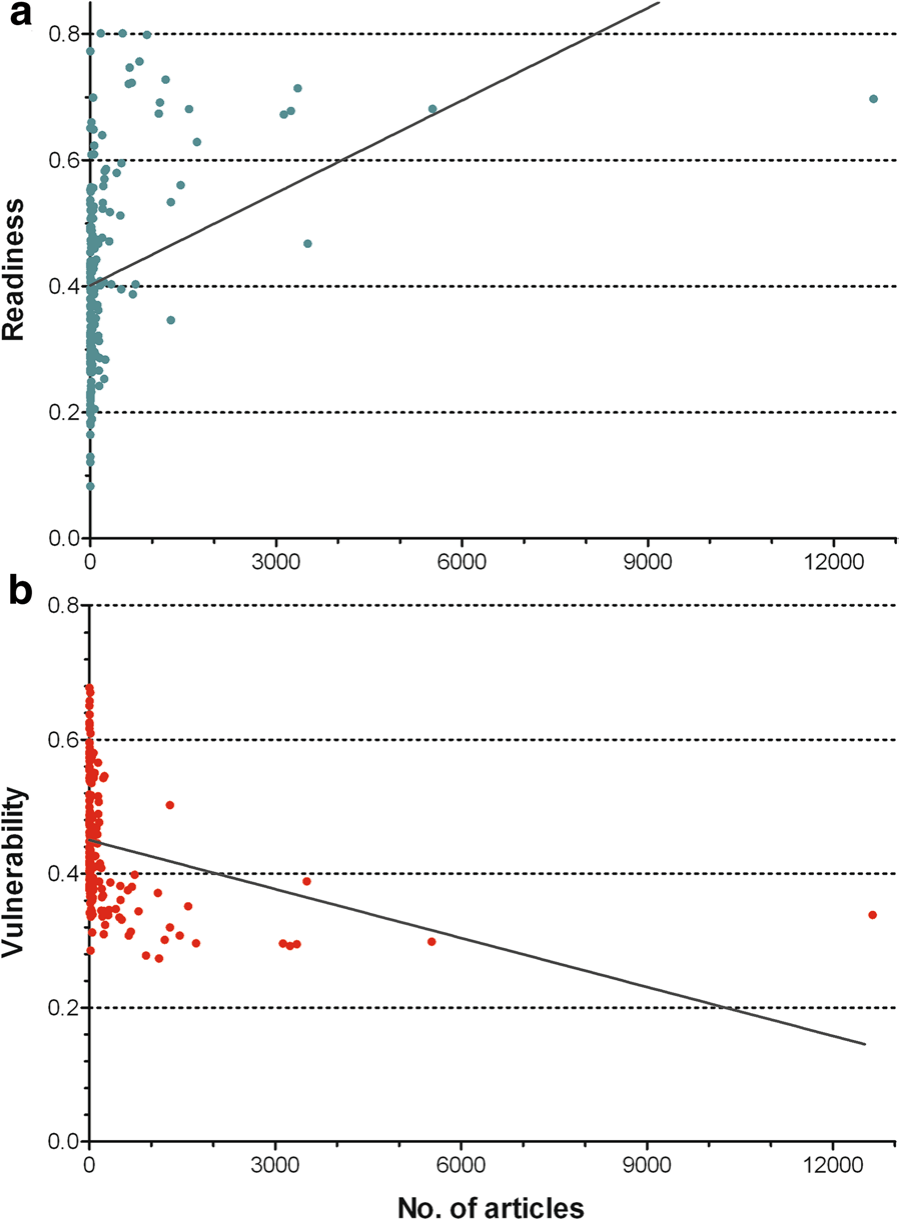
Correlation of the number of articles and indices of the ND-GAIN 2017 (Notre Dame Global Adaption Initiative) [27] regarding countries. **a** Readiness index, positive correlation (*p* < 0.001). **b** Vulnerability index, negative correlation (*p* < 0.001)

Correlation analysis of the two ND-GAIN indices (readiness and vulnerability) of 2017 and the number of articles were both significant (*p* < 0.0001), but with different slopes. The correlation of the readiness index and the number of articles was significantly positive (Fig. 9a), and the correlation of the vulnerability index and the number of articles was significantly negatively correlated (*p* < 0.001) (Fig. 9b).

### International networking

A total of *n* = 11,626 (29%) international cooperation articles were identified. Of these, *n* = 7995 were bilateral and *n* = 3165 trilateral collaborations, respectively. Four articles were worked out with at least 20 collaboration countries.

The first international cooperation in our database was published in 1975. Over time, the number of international partnerships increased exponentially, similar to the total number of articles, until it reached its maximum in 2014 with *n* = 1425 international collaboration articles.

**Fig. 10:**
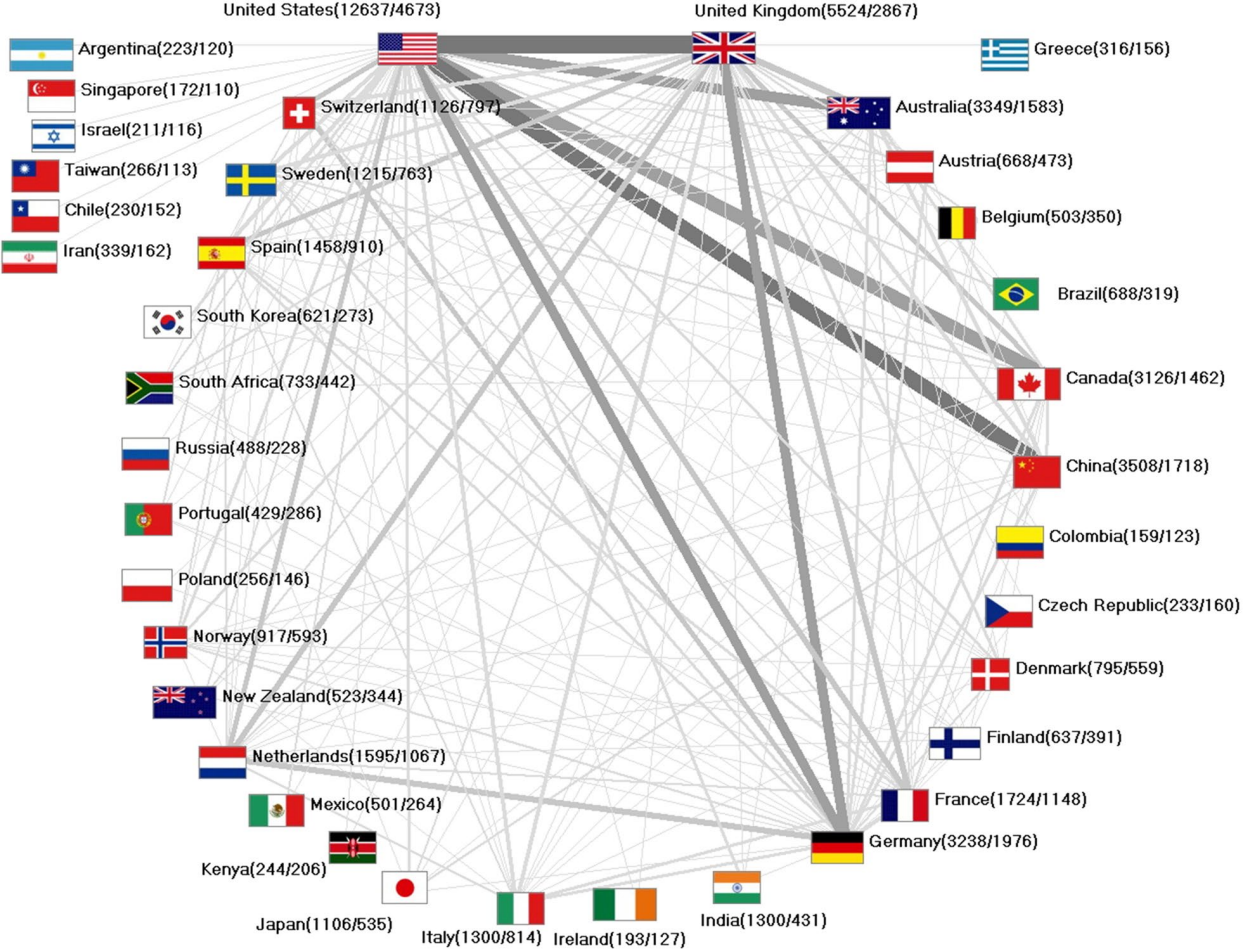
Network of internationally co-authored articles on climate change with numbers in brackets (number of articles/number of cooperation articles). The width of connecting lines represents the quantity of common articles (threshold: 40 collaboration articles between countries)

The USA as core country of the international networking participated in the 5 strongest partnerships (Fig. 10): USA/UK (*n* = 905), USA/China (*n* = 830), USA/Canada (*n* = 722), USA/Australia (*n* = 563), and USA/Germany (*n* = 534). Of the US articles, 37% were international collaborations, while more than half of the British articles and almost half of the Canadian and Australian articles were developed in international collaboration. Germany even conducted more than 60% of its studies with another country.

## Discussion

### Progress of publications on climate change

The first article on climate change identified by our approach was published as early as 1910. It is an article published in *Nature* and asked the question of whether the Indian climate changed [20]. This early publication already addressed the causal link between climate change and anthropogenic influence. The author asked whether there are causal links of increased irrigation and forest loss, also in comparison to statements by Gilbert Walker, the General Director of Indian Observatories, who made connections between the air pressure in South America and the intensity of Monsoon in India, thus negating links between climate change in India and human interference.

In 1947, an English article raised the question of whether there was a connection between the retreat of glaciers and climate change [4].

In 1956, the *carbon dioxide theory* was confirmed by a US-American article, which referred to a series of articles published as early as the end of the nineteenth century [31]. The authors of these articles formulated the carbon dioxide theory and thus provided the most widely accepted explanation for the climate change already recognized at that time. However, this was later denied until it turned out to be true. In his study, Gilbert N. Plass from John Hopkins University has already seen the impact of human activities on the CO_2_ balance through the combustion of fossil fuels, deforestation, and land management. In contrast to today’s threat awareness, the problem he discussed was the risk of new glacial formation due to the decrease of CO_2_ caused by a changed balance in the atmosphere–ocean system [31].

A German article from 1961 also argues that "man-made effects on climate change “should not be underestimated as well as "the danger that such effects will work irreversibly against human benefit” [11].

A study on the *astronomical theory*, also known as the *Milankovitch hypothesis* of climate change, raised in 1969 the problem awareness of the scientific world with its Barbados data [23].

In 1988, the *Intergovernmental Panel on Climate Change* (IPCC), an intergovernmental body of the United Nations (UN), was established at the first world climate conference in Geneva with the aim to provide a wide range of scientific information on climate change to support governmental decisions [16]. As of this first world climate conference, the number of publications has risen firstly to a three-digit figure, which can also be seen from the sharp increase in relative numbers per 10,000 SCI articles.

Thereafter, the numbers increased steadily until 2003, when an exponential increase could be observed that was also reflected by the steep rise in relative numbers. At the COP in Milan (Italy) in 2003, all parties agreed to the Adaption Fund, which was primarily founded to support developing countries in their capacity to respond to the consequences of climate change. In the same year, an enormous heatwave caused many thousands of deaths in Europe [37]. Since European countries, in particular, are among the most publishing nations, this regional climate catastrophe has certainly contributed to a strong increase in research interest on climate change.

Also, in 2003, the most cited article of this study was published. By analyzing more than 1700 species, the meta-analysis of C. Parmesan and G. Yohe shows that biological trends are in line with predictions of climate change [30]. This successful publication certainly contributed to the fact that the highest average citation rate per year was achieved in 2003, initiating an exponential growth in publication output.

The citation numbers increased adequately to the publication numbers with some outstanding years, e.g., 1991, 2000, 2004, and 2010, latter the year with the highest number of citations so far. Many of the high impact articles are published in these years so that an association can be assumed.

### Geographical aspects of publications on climate change

The USA, the UK, China, Australia, and Germany could be identified as the most publishing countries on climate change. This is not surprising, as it shows that mostly scientifically well-structured countries conduct most of the research, not only on climate change issues, as previous studies also have shown [17]. China, in particular, was catching up in the recent years due to its targeted research policy, which is represented by the enormous increase in expenditures on R&D [28].

The USA government, which is the most publishing country on climate change so far, is not exactly famous for its climate change–conscious attitude. The rejection of binding targets and the denial to sign the Paris Agreement confirms this. The USA is still the country with the highest expenditures on R&D and certainly one of the most preferred places to work for the most renowned scientists in the world. Despite the government’s attitude, its leading position in terms of publication output is not unique for climate change research and certainly not astonishing.

The results also show a clear dominance of European countries in the publication numbers on climate change. Also, Europe has a very good scientific infrastructure at its disposal. In contrast, most European countries signed up to the binding targets of the Paris Agreement to reduce emissions by at least 40% by 2030 compared to 1990 [10]. Denmark event targeted for a 70% reduction [7].

To evaluate the scientific landscape on climate change in greater depth, we extended the analyses to other, more differentiated parameters.

The Scandinavian countries have to be highlighted due to their leading position concerning various additional evaluation parameters, e.g., socioeconomic ratios. In general, Scandinavian countries have established good conditions for researchers and spend a lot on R&D. This is why research on climate change has also proven to be no exception. Sweden and Norway were leading in the analysis of their publication numbers in terms of national CO_2_ emission, with Switzerland in between in 2nd place. This parameter had been chosen for the analysis in order to establish a link with countries’ obligations under the polluter-pays principle. In 2017, the highest emissions rates were released by China, USA, India, Russia, Japan, Germany, Iran, and Saudi Arabia. Sweden ranks first when putting the number of published articles in relation to the emission rate (threshold 300 articles), followed by Switzerland, Denmark, Norway, and New Zealand.

The Scandinavian countries are known to be “early adopters of renewable energy”. The share of renewable energy in Iceland is 77%, in Sweden 63%, in Norway 51% (despite the oil production capacity), in Finland 47%, and in Denmark 33%, in contrast to the EU28 with a proportion of only 21% [26]. Also, in terms of the relation to the number of people living on exposed land to sea-level rise, Sweden led in the evaluation of absolute numbers and Finland of relative numbers. With 1215 articles, Sweden ranked 12th regarding its absolute publication numbers, Norway 15th, Denmark 16th, and Finland 20th.

Switzerland, which is ranked second in terms of inclusion of CO_2_ emission, is affected to a considerable extent by climate change due to its location in the European Alps and the progressive melting of glaciers and permafrost. Especially since tourism—above all skiing—is an important economic sector. Therefore, it is not astonishing that the focus of Swiss research is mainly on problems related to the Alpine region [6].

In terms of science-related parameters, such as GERD or number of researchers, both Australia and New Zealand came into focus. Since they are located close to each other, the intensity of cooperation in climate change research is understandable. The location near Antarctica on the one hand and the immense heatwaves with extraordinary effects on ecosystems and biosphere on the other hand form the background for relatively high investments in climate change research.

Looking at the average citation rate of the publishing countries (*n* ≥ 30), Costa Rica occupied a prominent position. With 67 articles, Costa Rica is far behind in absolute terms. Nevertheless, these articles were cited 6291 times. Nearly half of the studies of Costa Rica are worked in collaboration with the USA. The Tropical Science Center (TSC) affiliated with the Monteverde Cloud Forest Biological Reserve in Costa Rica participated in a US-American and Costa Rican collaboration, with the 5th most cited article of this analysis, that deals with the impacts of climate change on wildlife [34]. The Center is also taking part in two other high-profile publications, which, like the most cited article, are also published in *Nature*. They all deal with the risk of extinction caused by global warming. Alan Pounds, biological scientist since 1996 at the TSC and focusing on the biological impact of climate change, found, e.g., a correlation between amphibian die-offs and rising average temperatures [32]. Previously, he worked at the Department of Zoology, University of Florida, USA, where he already collaborated with colleagues from Costa Rica. The pattern of the successful partnership of international networks can be seen in this example, which stands for mutual benefit for both cooperating countries.

In terms of citation rates, Estonia also took a leading position, as it is part of a Europe-wide meta-analysis on changes in phenology using data on more than 125,000 observational series of plants and animals to assess their response to climate change [22]. The resulting article, which was published in 2006 in Global Change Biology, received almost half of the Estonian citations.

The third country that should be highlighted regarding the citation rate of its articles is Iceland. Its articles were not counted among the high-impact publications. Instead, many of its articles achieved recognition with above-average citation rates. The location far in the north, close to Greenland and the Arctic Circle, is an advantage for all climate change projects that focus on melting glaciers in these regions, and the glacial retreat is here more and more evident. It has been assumed that all Islandic glaciers will be disappeared by the year 2200 [26]. Therefore, the most cited Icelandic article is the result of an international collaboration focusing on the regional differences in the last glacial period to better understand climate dynamics [3].

The comparison of the countries’ results in relation to the GDP put the insular state of Fiji, which consists of more than 300 islands, at the top of the evaluation. Currently, almost one million people are living in an area of about 18,000 square kilometers north of New Zealand in the South Pacific. Fiji has been selected to chair the 23rd climate summit 2017 in Germany. In the same year, the number of articles from Fiji reached its maximum, which seems to be associated. Nearly half of the articles are collaboration works with Australia. One of the advantages of research cooperation on climate change is the existence of Fiji’s coral reefs, their vulnerability, and their importance for coastal protection.

Worthy to note is also the rank of Denmark in terms of socioeconomic influence. In addition to Denmark’s otherwise equally good scientific infrastructure, its position in climate change research is certainly influenced by Greenland’s affiliation and the direct and immediate effects of climate change in this region located closest to the Arctic. The direct association to Greenland or the Arctic can be found in more than 200 Danish articles mostly focusing on *Geoscience*. The Niels Bohr Institute at the University of Copenhagen is leading in the climate change research based on ice cores. The ice core collection is considered as a “national treasure” and contains a deep drill core of more than 15 km in length [43].

In connection with the socioeconomic analysis, it is also remarkable that the African developing country Zimbabwe ranked 7th among the top 10. Unlike other African countries, it is relatively industrialized and produces twice the average amount of greenhouse gases [5]. Nevertheless, Zimbabwe—like other African countries—has to cope with droughts, freshwater and food shortages, diminished biodiversity, vector-borne diseases, and dry ups as a result of climate change. Zimbabwe was among the first countries to sign and ratify the *UN Framework Convention on Climate Change* (UNFCCC) in 1992 [29, 41]. In 2011, it participated in the REDD+ program (*Reducing Emissions from Deforestation and Forest Degradation*), which aims to avoid 52 million tons of CO_2_ over 30 years in Zimbabwe and in return to support the communities with financial aid for agriculture, fire prevention, and production methods to preserve forest areas [5].

France, which ranks 7th in terms of absolute publication numbers, led when the ratio between publication numbers and fatalities due to climate events of the CRI index is assessed. More than half of the articles on climate change are worked out with the participation of the French state research organization *Centre National de la Recherche Scientifique* (CNRS). The CNRS was ranked 4th by the Nature Index in 2017 regarding the largest contributors, behind the CAS (in this study identified as most publishing institute on climate change), Harvard University USA, and Max Planck Society Germany [25]. Plus, the majority of its articles is worked out as international collaboration (66.59%). The share of the other most publishing countries is considerably lower: USA (36.98%), UK (51.90%), China (48.97%), Australia (47.27%) Germany (61.10%), Canada (46.77%).

Nevertheless, the share of collaboration articles is relatively high in comparison to other research fields. This may be due to the majority of articles published after 2000, considering that the share of collaboration articles generally increases over time due to the international awareness of its benefits [1].

## Conclusions

Articles on climate change focused on three main thematic groups, leading from the modeling of future scenarios to the environmental and socioeconomic impacts and the corresponding mitigation and adaptation measures. The readiness of countries and their vulnerability are inversely related to the number of articles published on climate change. Our results show the dominance of the Northern hemisphere in terms of publication output on climate change. Taking into account socioeconomic, research, and climate-specific characteristics, the order of the leading countries shifts, but the main actors remain the same with only a few exceptions. Only Costa Rica, Fiji, and Zimbabwe as developing countries came to play a role in the evaluation of the results. In principle, Africa, Asia, and South America are extremely under-represented. Many scientists are becoming aware of the advantages of international networking, which is of mutual benefit to all participating countries. However, particularly regarding climate change research, these benefits should be more frequently shared with developing countries, as the involvement of these most affected nations still is sparse.

In this context, the term “equity” is certainly familiar to all those interested in climate change research. There is a heated debate in the scientific community on whether scientific cooperation with developing countries should be called for. Many researchers see this as limiting the freedom of research. However, the principle of research responsibility should also be taken into account in this context. This should or must lead to a global risk-indexed joint planning because all scientists have only one planet to take care of. In this context, Prof. Drenth, Emeritus, Psychometrics and Organizational Psychology, Free University Amsterdam [8], asked the following questions for any scientist dealing with climate change: “Risks for whom? How far does the right to know go? What is the balance between self-determination and the interests of larger groups or the society as a whole? How certain does the scientist have to be before warning, especially in the case of irreversible developments?”.

The spread and economic impact of the current COVID 19 pandemic has reduced public and media interest in climate change issues. All the more reason to urgently press for the causes and consequences of climate change to once again become the focus of interest, while at the same time dealing with the consequences of the pandemic. Climate change must continue to be recognized as one of the most urgent global challenges. This makes it necessary to reconcile future scientific direction with the long-term environmental, social and economic consequences of the impacts of climate change that all countries are facing.

## Data Availability

The bibliometric data are the property of the Web of Science database and were obtained from it. Therefore, the authors are not allowed to pass on the data publicly or privately. Any researcher with access to the Web of Science database can obtain the data using the methods described in the paper. Readers who do not have access to Web of Science should contact Clarivate Analytics to obtain a license.
